# Bioinformatic analysis of the obesity paradox and possible associated factors in colorectal cancer using TCGA cohorts

**DOI:** 10.7150/jca.80977

**Published:** 2023-01-22

**Authors:** Dong Min Lim, Hyunsu Lee, Kisang Eom, Yun Hak Kim, Shin Kim

**Affiliations:** 1 Interdisciplinary Program of Genomic Data Science, Pusan National University, Yangsan 50612, Korea.; 2 Department of Medical Informatics, School of Medicine, Keimyung University, 1095 Dalgubeol-daero, Dalseo-gu, Daegu 42601, Republic of Korea.; 3 Department of Physiology, School of Medicine, Keimyung University, 1095 Dalgubeol-daero, Dalseo-gu, Daegu 42601, Republic of Korea.; 4 Department of Biomedical Informatics, School of Medicine, Pusan National University, Yangsan 50612, Republic of Korea.; 5 Department of Anatomy, School of Medicine, Pusan National University, Yangsan 50612, Korea.; 6 Department of Immunology, School of Medicine, Keimyung University, Dalseo-gu, Daegu 42601, Republic of Korea.; 7 Institute of Medical Science, Keimyung University, Dalseo-gu, Daegu 42601, Republic of Korea.; 8 Institute for Cancer Research, Keimyung University Dongsan Medical Center, Dalseo-gu, Daegu 42601, Republic of Korea.

**Keywords:** Obesity paradox, colorectal cancer, TCGA, tumor-infiltrating immune cells, intratumoral microbiota

## Abstract

Colorectal cancer (CRC) is a common malignancy worldwide and the second leading cause of cancer-related deaths. Obesity is an important determinant of CRC incidence; however, obese patients have also shown better long-term survival than non-obese patients, suggesting that the development and progression of CRC are associated with different mechanisms. This study compares the expression of genes, tumor-infiltrating immune cells, and intestinal microbiota between high- and low-body mass index (BMI) patients at the time of CRC diagnosis. The results revealed that high-BMI patients with CRC have better prognosis, higher levels of resting CD4+ T cells, lower levels of T follicular helper cells, and different levels of intratumoral microbiota than low-BMI patients. Our study highlights that tumor-infiltrating immune cells and intratumoral microbe diversity are major features of the obesity paradox in CRC.

## Introduction

Colorectal cancer (CRC) is the third-most diagnosed cancer worldwide and the second leading cause of cancer-related deaths. According to Xi and Xu 1, the proportion of CRC incidence and related deaths are expected to increase substantially by 2040 because of the impacts of a westernized diet and modern sedentary lifestyles. Although obesity is considered to be an important risk factor of CRC 2, 3, contradictory reports have been obtained regarding its role in CRC progression, and it has also been associated with increased survival rates in CRC patients 4-6.

Being overweight or obese is known to increase the risk for various chronic diseases, such as cancer or cardiovascular disease 7, 8. However, in various consumptive chronic diseases - several cancers and tuberculosis - lower body mass index (BMI) has been associated with worse prognosis 9-12. This phenomenon in which the prognosis of overweight patients is superior to that of underweight and normal weight patients is known as the “obesity paradox” 13. Although obesity was associated with greater overall mortality in cancer patients, obese patients with lung cancer, renal cell carcinoma, melanoma, and CRC had better prognoses than under- or normal weight patients with the same conditions 14-20. Given that obesity is a confirmed risk factor for CRC and metabolic syndromes 21, the mechanism underlying the obesity paradox in cancer remains ambiguous 22.

Tumor-infiltrating immune cells (TIICs) play an important role in tumor development and determining clinical outcomes 23. TIICs are promising biomarkers for the diagnosis and prognosis of non-metastatic CRC 24, and have achieved greater prognostic performance than histopathological methods 25. We hypothesize that the evaluation of TIICs could elucidate the molecular mechanisms associated with the obesity paradox in CRC.

The gut microbiome plays a crucial role in the local and systemic immunomodulation of various diseases, including tuberculosis, cardiovascular disease, and cancer 26-28. A previous report showed a strong relationship between the intake of certain bacteria and the inhibition of colon cancer progression 29, which is achieved via intestinal homeostasis and immune regulation 30, 31. Although the relationship between obesity and gut microbiota composition has been widely investigated 32, the complex and dynamic relationships between gut microbiota, obesity, and CRC remain unclear.

This study describes the obesity paradox of CRC in cohorts of The Cancer Genome Atlas (TCGA) and investigates the associated characteristics of TIICs and intratumoral microbiome using whole genome and RNA sequencing analyses.

## Materials and Methods

### Data acquisition and pre-processing

Gene expression, methylation, and clinical data were downloaded from TCGA-COAD (colorectal adenocarcinoma) and TCGA-READ (rectal adenocarcinoma) databases. Data are available at the Genomic Data Commons data portal 33 (https://portal.gdc.cancer.gov). Overall survival (OS) data were downloaded from the UCSC Xena Browser 34 (http://xena.ucsc.edu). BMI was calculated as weight divided by height squared (kg/m^2^) and categorized based on WHO classifications (high-BMI group, BMI ≥ 30; low-BMI group, BMI ≤ 25). Asian patient data were excluded from TCGA cohorts because the Asia-Pacific BMI classification differs from that of WHO. We investigated the intratumoral microbiome using the Kraken analysis, as previously described 35. The Kraken algorithm is a rapid and highly accurate program for assigning taxonomic labels to metagenomic sequences using k-mers alignment 36. We downloaded microbiome data for CRC patients from an online data repository (ftp://ftp.microbio.me/pub/cancer_microbiome_analysis/TCGA/Kraken/). We specifically used intratumoral microbiome abundance (“Kraken-TCGA-Voom-SNM-Plate-Center-Filtering-Data.csv”) and clinical data (“Metadata-TCGA-Kraken-17625-Samples.csv”). Pre-processing of data was performed using R software (v4.1.1) 37. Patient information is listed in Table 1.

### Differentially expressed gene (DEG) analysis

We used the edgeR package (v3.34.1) in R to identify DEGs 38. First, DEGs were identified for primary tumor samples of the high- and low-BMI patient groups. We used the Benjamini-Hochberg adjusted *P* < 0.05 and |log_2_ fold change| > 0.05 to identify genes with upregulated and downregulated expressions. We filtered unexpressed and low counts using the edgeR function filterByExpr, with the minimum count required for at least some samples = 10, minimum total count required = 15, and minimum proportion of samples in the smallest group that expressed the gene = 0.7. The trimmed mean of the M-values were normalized and analyzed using the edgeR function glmQLFTest. A quasi-likelihood test, which fits the data to a quasi-likelihood negative binomial generalized log-linear model, was used to perform gene-specific analyses for a given coefficient or contrast. The EnhancedVolcano package (v1.10.0) in R was used to visualize the DEGs 39.

### Protein-protein interaction (PPI) network construction and module selection

To identify the pathways and functions associated with the DEGs, PPI networks were constructed using the Search Tool for the Retrieval of Interacting Genes (STRING) database (http://www.string-db.org) 40 and Cytoscape software (v3.8.2) 41. The Molecular Complex Detection (MCODE) plugin was used for module selection with the following parameters 42: degree cutoff, 2 × cluster finding; node score cutoff, 0.2 × K-core = 2 ×; maximum depth, 100.

### Functional analysis of DEGs

DEG pathways were further assessed by gene ontology (GO) and Kyoto Encyclopedia of Genes and Genomes (KEGG) enrichment analyses using the ClusterProfiler package (v4.0.5) in R 43, and GO and KEGG enrichment analyses were performed using the enrichment plot (v1.12.3) R package 44. Significance was set at *P* < 0.05.

### Analysis of TIICs based on machine learning

We estimated the proportions of infiltrating immune cell types in each sample using bulk RNA sequencing data, which was analyzed using the CIBERSORTx algorithm 45. CIBERSORTx is a machine learning algorithm that extends the CIBERSORT framework to infer cell-type-specific gene expression profiles without physical cell isolation. We included 22 immune cell subtypes parsed from the gene signature matrix LM22 and 1,000 permutations of the CIBERSORTx web portal (http://cibersortx.stanford.edu/) with bulk-mode batch correction.

### Linear discriminant analysis effect size (LEfSe)

To identify unique microbial signatures in the CRC samples between the high- and low-BMI patients, we analyzed the Kraken-TCGA datasets using a linear discriminant analysis in the Galaxy web application (http://huttenhower.sph.harvard.edu/galaxy) 46. To identify taxa with significantly differential abundance, we used the factorial Kruskal-Wallis test for classes and pairwise Wilcoxon test for subclasses, with the significance set at *P* < 0.05. The threshold for the logarithmic LDA score for discriminative features was set at 2.

### Differential methylation probes (DMPs)

Methylation data (downloaded from TCGA database) included 159 CRC patients (79 high and 80 low BMI samples). We used the ChAMP package (v2.24.0) to identify the DMPs between the high- and low-BMI groups 47. A beta-mixture quantile normalization was performed to correct the probe design bias in Illumina 450k DNA methylation data. Batch effects were corrected using the ChAMP function ComBat to reduce technical variation. We selected the cutoff values *P* < 0.05 and |deltaBeta| > 0.05 to define hypermethylated and hypomethylated genes.

### Statistical analysis

OS rate was compared between the high- and low-BMI groups using Kaplan-Meier survival curves and a log-rank *P*-value. Hazard ratios (HRs) and 95% confidence intervals (CIs) were estimated using a Cox proportional hazards model to investigate the association between patient survival and multiple predictors. A Schoenfeld individual test was performed to confirm that the assumptions of the Cox proportional hazards model were met (Supplementary Figure S1). The Wilcoxon signed-rank test was used to compare the mean difference in the immune cell fractions of CRC patients. Venn diagrams were generated using Venny software 48. All statistical analyses and visualizations were performed using R software 37.

## Results

### BMI and patient survival

**Figure 1:**
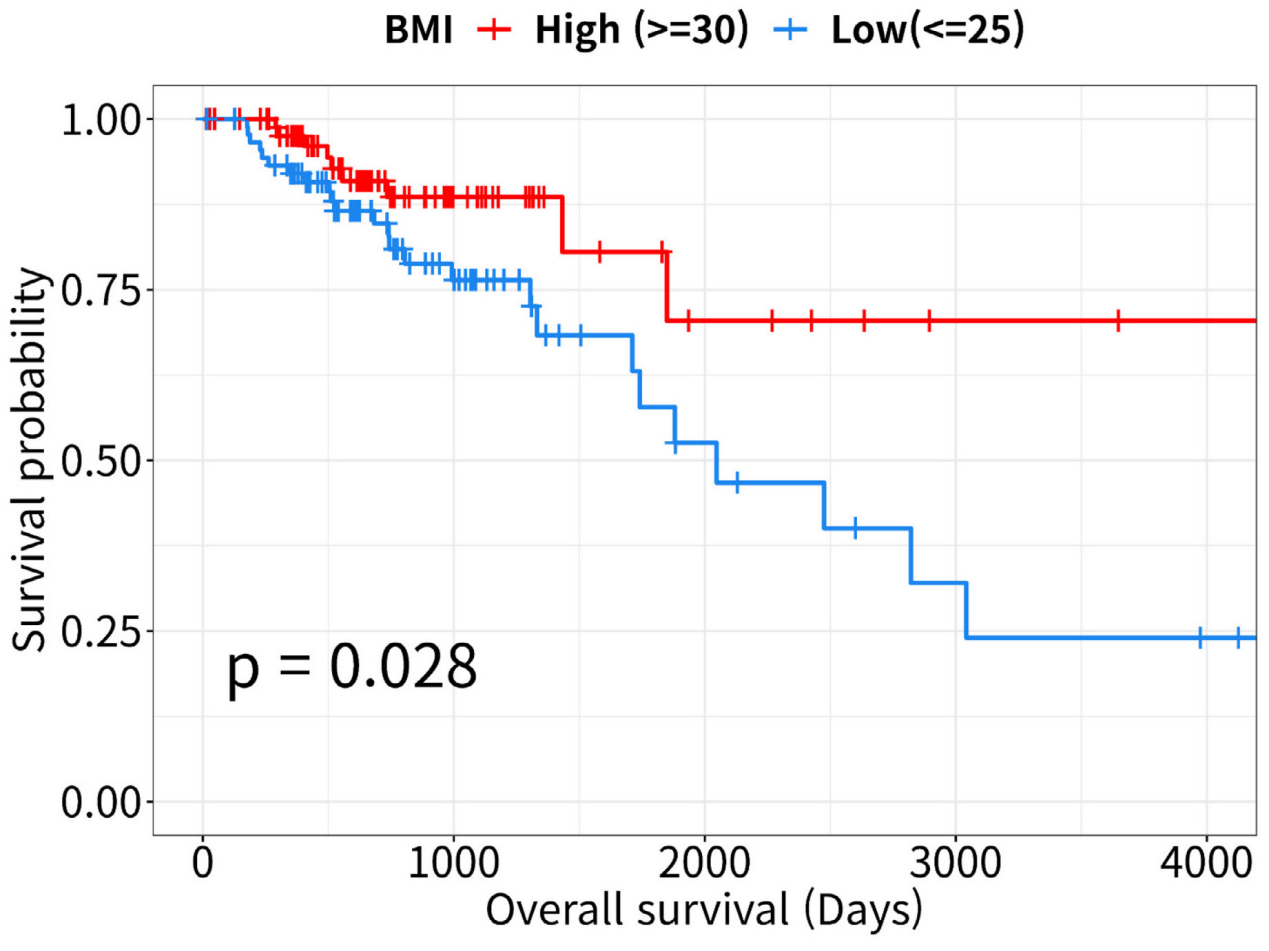
** Kaplan-Meier curves for overall survival analysis.** Survival curves of high- and low- body mass index (BMI) patients with colorectal cancer (CRC, n = 178). Data were downloaded from the UCSC Xena Browser (http://xena.ucsc.edu).

The Kaplan-Meier survival curves revealed that OS rates were lower in the low-BMI than in the high-BMI CRC patient group (log-rank *P* = 0.028, Figure 1). According to the Cox proportional hazards model, patient survival in the low-BMI group was significantly affected by age, sex, and TNM stage (HR = 2.49, 95% CI: 1.06-5.9, *P* = 0.037; Table 2).

### Molecular differences between high- and low-BMI groups in CRC patients

**Figure 2:**
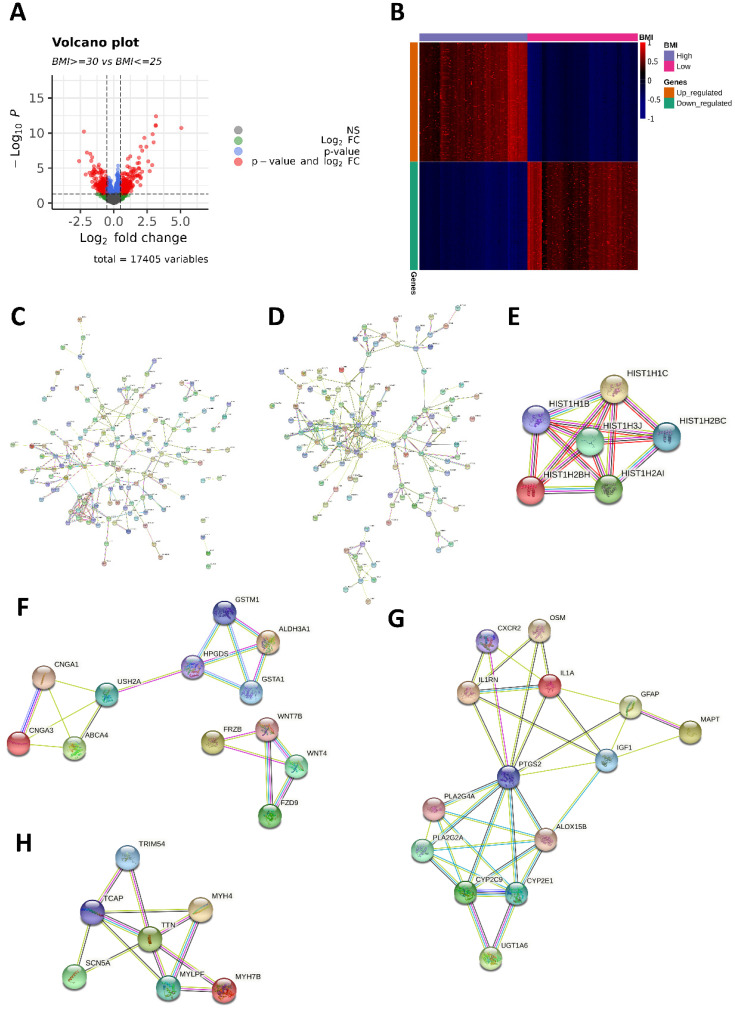
** Differentially expressed genes (DEGs) and protein-protein interaction (PPI) network. (A)** Volcano plot of the 569 DEGs (298 upregulated and 271 downregulated in the high-BMI group) between high- and low-BMI patients with CRC. Genes corresponding to the Benjamini-Hochberg adjusted *P* < 0.05 and log_2_ fold change > 2 are denoted in red; genes corresponding only to the adjusted *P* < 0.05 are denoted in blue; genes corresponding only to the log_2_ fold change > 2 are denoted in green; and genes not corresponding to either the p-value or log_2_ fold change are denoted in black.** (B)** Heatmap of 569 DEGs between high- and low-BMI patients with CRC based on the normalized Z-score. **(C)** PPI network of 298 genes with upregulated expressions. **(D)** PPI network of 271 genes with downregulated expressions. The module with the **(E)** highest and **(F)** second-highest Molecular Complex Detection (MCODE) value for the genes with upregulated expressions. The module with the **(G)** highest and **(H)** second-highest MCODE value for the downregulated genes.

We identified 569 DEGs (298 upregulated and 271 downregulated in the high-BMI group) between the high- and low-BMI groups. Volcano plots and a heatmap of the 569 DEGs are shown in Figure 2A and 2B. From the PPI network (Figure 2C-D), we identified significant modules of genes with upregulated and downregulated expressions using MCODE. In the PPI network of the 298 genes with upregulated expressions, the module with the highest MCODE score contained six genes (*HIST1H1B*, *HIST1H1C*, *HIST1H2AI*, *HIST1H2BC*, *HIST1H2BH*, and *HIST1H3J*; Figure 2E). The second-most significant module comprised 12 genes (*ABCA4*, *ALDH3A1*, *CNGA1*, *CNGA3*, *GSTA1*, *GSTM1*, *HPGDS*, *USH2A*, *FRZB*, *FZD9*, *WNT4*, and *WNT7B*; Figure 2F). In the PPI network of the 271 genes with downregulated expressions, the most significant module comprised 14 genes (*ALOX15B*, *CXCR2*, *CYP2C9*, *CYP2E1*, *GFAP*, *IGF1*, *IL1A*, *IL1RN*, *MAPT*, *OSM*, *PLA2G2A*, *PLA2G4A*, *PTGS2*, and *UGT1A6*; Figure 2G) and the second-most significant module comprised seven genes (*MYH4*, *MYH7B*, *MYLPF*, *SCN5A*, *TCAP*, *TRIM54*, and *TTN*; Figure 2H).

### Functional enrichment analysis of DEGs

**Figure 3:**
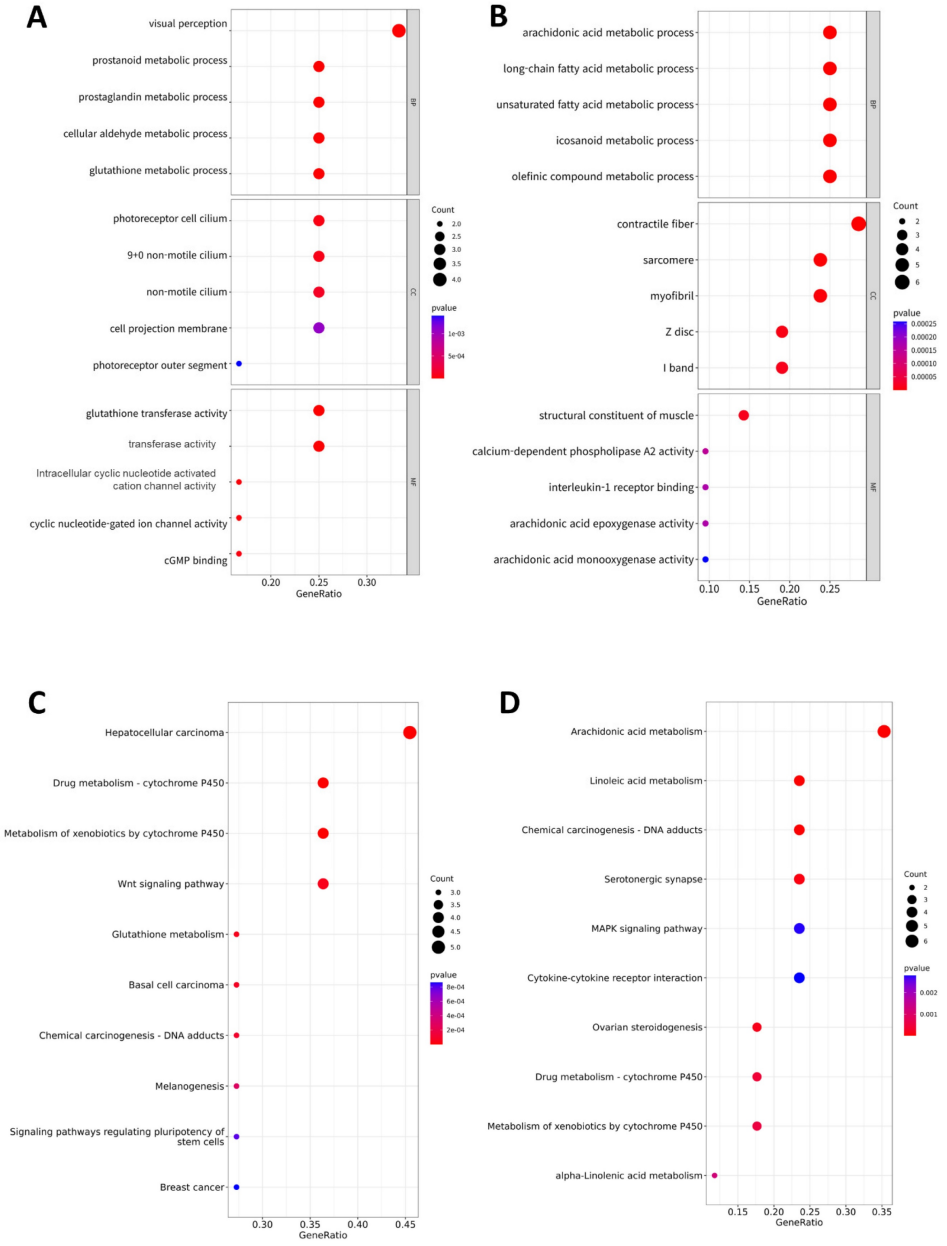
** Functional analysis.** Gene ontology (GO) assay for the 39 genes comprising the significant modules in the PPI network: **(A)** 18 upregulated genes and **(B)** 21 downregulated genes. GO analysis considered the biological process (BP), cellular component (CC), and molecular function (MF) terms. Dot plots show the results corresponding to *P* < 0.05. Size of the circle indicates the number of genes corresponding to each term. The closer to red, the lower the p-value. Kyoto encyclopedia of genes and genomes (KEGG) assay of the 39 genes comprising the significant modules in the PPI network: **(C)** 18 upregulated genes and **(D)** 21 downregulated genes. The size of the circle indicates the number of genes corresponding to each term. Dot plots show the results corresponding to *P* < 0.05. The closer to red, the lower the p-value.

To identify the functional factors of the highly networked protein groups, we performed GO and KEGG enrichment analyses to determine the biological roles of the 39 genes (upregulated: 18, downregulated: 21) comprising the significant modules in the PPI network (Figure 3). The GO analysis considered three major categories: biological process (BP), cellular component (CC), and molecular function (MF). The top GO categories of the 18 genes with upregulated expressions were visual perception, prostanoid metabolic process, prostaglandin metabolic process, photoreceptor cell cilium, and glutathione transferase activity (Figure 3A). The top GO categories of the top 21 genes with downregulated expressions were arachidonic acid (AA) metabolic process, long-chain fatty acid metabolic process, unsaturated fatty acid metabolic process, contractile fiber, and structure constituent of muscle (Figure 3B). According to the KEGG pathway analysis, the 18 genes genes with upregulated expressions were primarily associated with hepatocellular carcinoma, cytochrome P450, Wnt signaling pathway, glutathione metabolism, basal cell carcinoma, DNA adducts, melanogenesis, signaling pathways regulating pluripotency of stem cells, and breast cancer (Figure 3C); the 21 genes with downregulated expressions were primarily associated with AA metabolism, linoleic acid metabolism, DNA adducts, serotonergic synapse, MAPK signaling pathway, cytokine receptor interaction, ovarian steroidogenesis, metabolism of cytochrome P450, and alpha-linolenic acid metabolism (Figure 3D).

### Investigation of DNA methylation-driven DEGs

**Figure 4:**
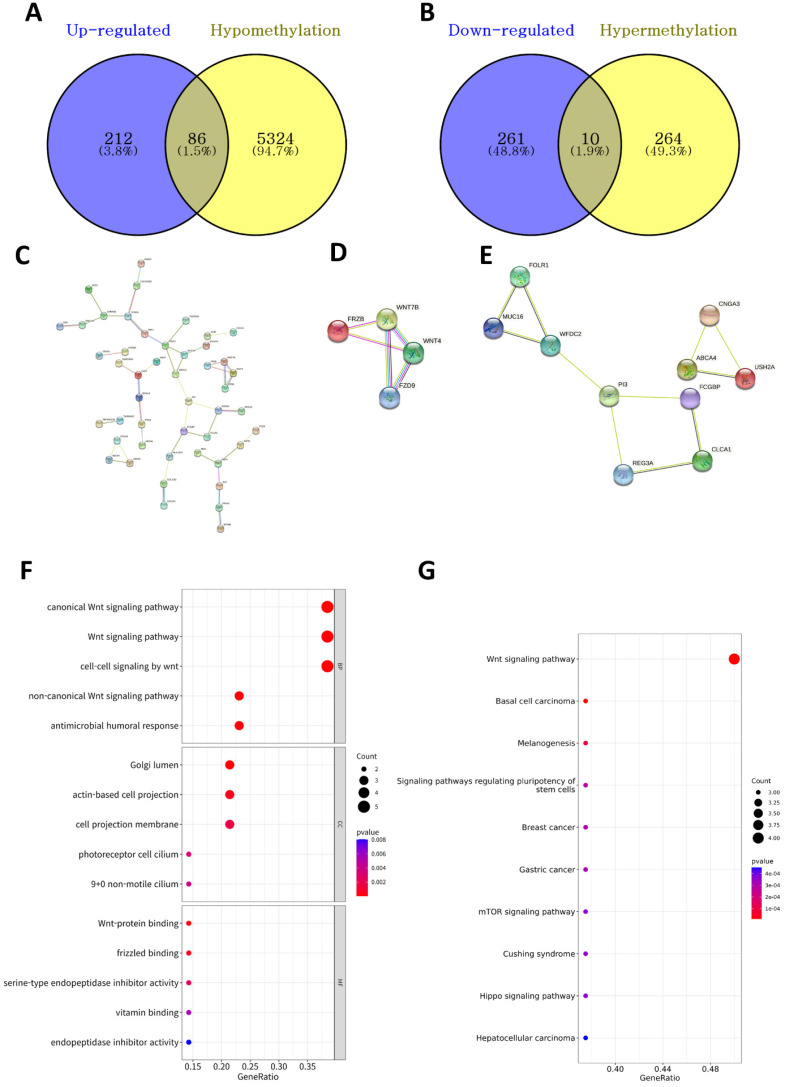
** Analysis of DNA methylation-driven DEGs. (A)** The Venn diagram shows the overlapping upregulated and hypomethylated DEGs. **(B)** The overlapping downregulated and hypermethylated DEGs. **(C)** PPI network of upregulated-hypomethylated genes. The module with the **(D)** highest and **(E)** second-highest MCODE value for upregulated-hypomethylated genes. **(F)** GO assay of 14 upregulated-hypomethylated genes comprising the module with the highest MCODE value. GO analysis considered the biological process (BP), cellular component (CC), and molecular function (MF) terms. Dot plots showing results corresponding to *P* < 0.05. Size of the circle indicates the number of genes corresponding to each term. The closer to red, the lower the p-value. **(G)** KEGG assay of 14 upregulated-hypomethylated genes comprising the module with the highest MCODE value. The size of the circle indicates the number of genes corresponding to each term. Dot plots showing results corresponding to *P* < 0.05. The closer to red, the lower the p-value.

We identified 5,684 DMPs (274 hypermethylated and 5,410 hypomethylated genes in the high-BMI group) between the high- and low-BMI groups. We investigated the overlapping DEGs (Figure 2A) and DMPs between the high- and low-BMI groups (Figure 4A and 4B) and identified 86 upregulated and hypomethylated genes and 10 downregulated and hypermethylated genes. Figure 4C shows the PPI network of the 86 upregulated-hypomethylated genes. The module with the highest MCODE contained four genes (*FRZB*, *FZD9*, *WNT4*, and *WNT7B*; Figure 4D); the module with the second-highest MCODE comprised 10 genes (*ABCA4*, *CLCA1*, *CNGA3*, *FCGBP*, *FOLR1*, *MUC16*, *PI3*, *REG3A*, *USH2A*, and *WFDC2*; Figure 4E). The PPI network of the 10 downregulated-hypermethylated genes did not show statistically significant results. The top GO categories of the 14 upregulated-hypomethylated genes were the Wnt signaling pathway, Golgi lumen, actin-based cell projection, and Wnt-protein binding (Figure 4F). In the KEGG enrichment analysis, the 14 upregulated-hypomethylated genes were associated with Wnt signaling pathway, basal cell carcinoma, melanogenesis, signaling pathways regulating pluripotency of stem cells, breast cancer, gastric cancer, mTOR signaling pathway, Cushing syndrome, Hippo signaling pathway, and hepatocellular carcinoma (Figure 4G).

### Differences in TIICs between high- and low-BMI groups in CRC patients

**Figure 5:**
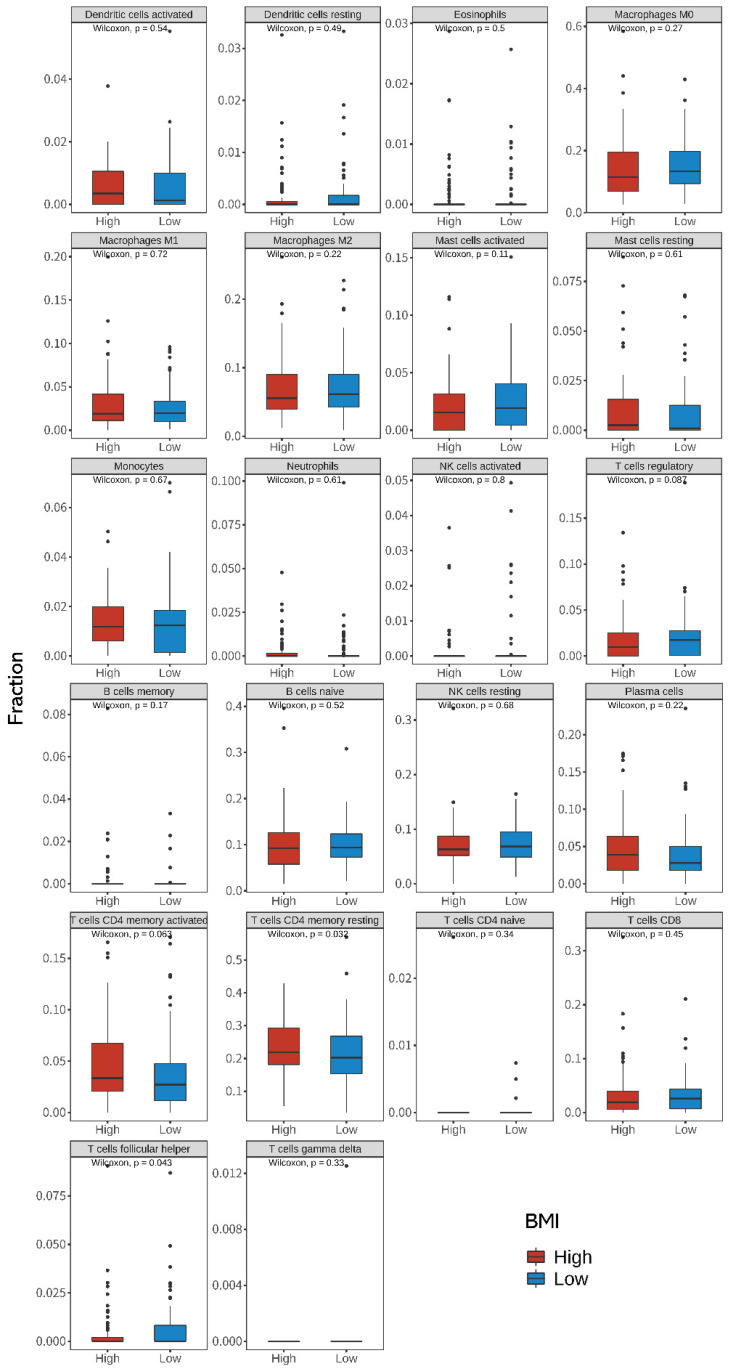
** Differences in the 22 immune cell fractions between high- and low-BMI patients with CRC.** Differences in the expression of tumor-infiltrating immune cells (TIICs) between the high- and low-BMI groups were evaluated using the Wilcoxon sign-rank test (two-sided). CRC, colorectal cancer; BMI, body mass index.

Using the CIBERSORTx algorithm, we estimated the relative abundance of 22 immune cells from the bulk tumor RNA sequencing data. Figure 5 shows the differences between the 22 TIICs according to the BMI groups. When compared with that in the low-BMI group, we found that resting CD4+ T cells were more abundant (*P* = 0.032, Wilcoxon's rank test) and T follicular helper (Tfh) cells were less abundant in the high-BMI group (*P* = 0.043, Wilcoxon's rank test). Spearman correlation analysis showed that *CNGA3*, *GSTA1*, *HPGDS*, *FRZB*, and *WNT4* were significantly correlated with resting CD4+ T cells. *IL1RN*, *OSM*, and *PLA2G2A* were significantly correlated with T follicular helper (Tfh) cells (Supplementary Figures S2 and S3). Univariate and multivariate Cox regression analyses between BMI and TIIC of CRC patients are shown in Supplementary Table S1.

### Investigation of unique microbial signatures between BMI groups

**Figure 6:**
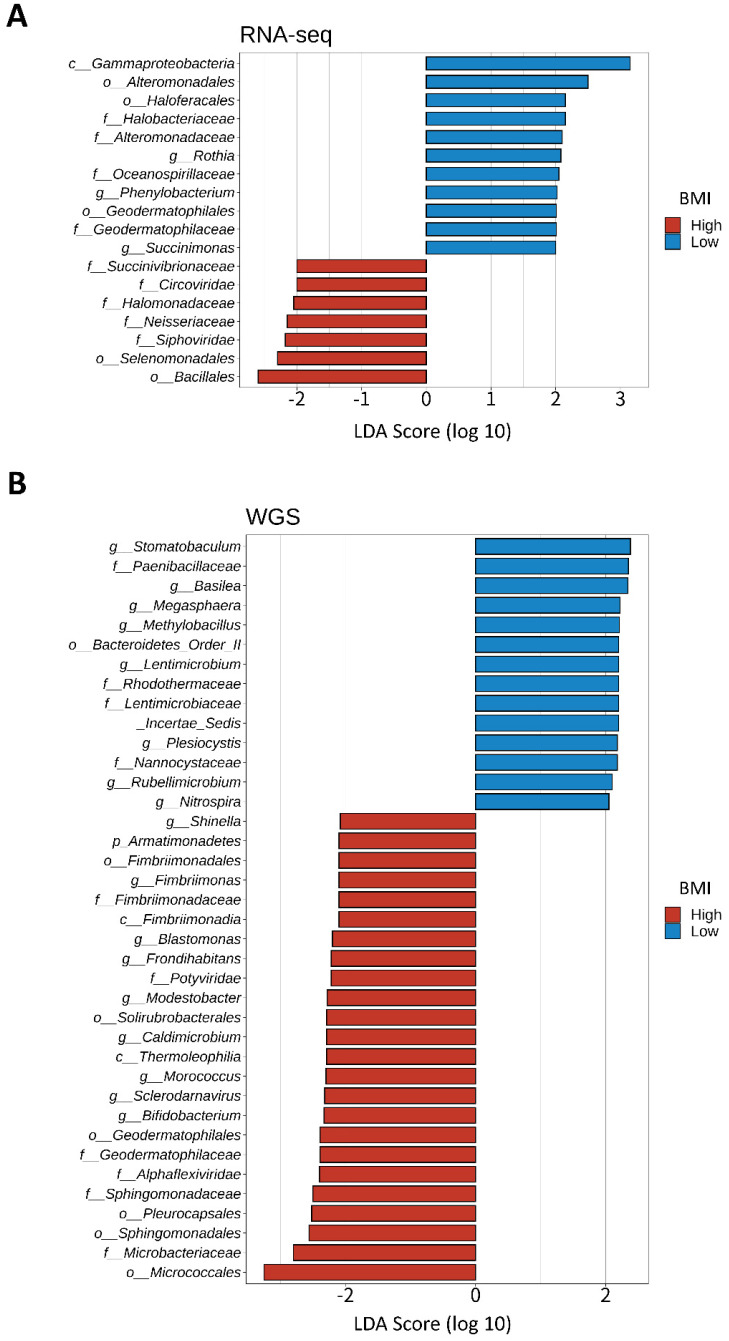
** LEfSe of the Kraken-TCGA dataset.** Linear discriminant analysis effect size (LEfSe) and distribution of the associated microbiota by subtype using RNA and whole genome sequencing (WGS).

We divided 184 CRC samples into 23 DNA whole genome sequencing (seven high- and 16 low-BMI samples) and 161 RNA sequencing samples (79 high- and 82 low-BMI samples). LEfSe of the Kraken-TCGA dataset identified nine enriched microbe genera in the high-BMI group (*Shinella*, *Fimbriimonas*, *Blastomonas*, *Frondihabitans*, *Modestobacter*, *Caldimicrobium*, *Morococcus*, *Sclerodarnavirus*, and *Bifidobacterium*; Figure 6A) and 11 enriched microbe genera in the low-BMI group (*Rothia*, *Phenylobacterium*, *Succinimonas*, *Stomatobaculum*, *Basilea*, *Megasphaera*, *Methylobacillus*, *Lentimicrobium*, *Plesiocystis*, *Rubellimicrobium*, and *Nitrospira*; Figure 6B).

## Discussion

Given the high incidence and mortality rates for CRC globally, intensive efforts are being made to discover effective prognostic factors and elucidate the molecular mechanisms of CRC to improve patient prognosis 5, 6, 49-51. Although controversial, the obesity paradox - first noted with the high survival rates of hemodialysis patients with high BMI 52 - has been reported in various chronic diseases 53-56, including CRC 57-62. However, bioinformatic studies on the characteristics of TIICs and intratumoral microbiome associated with the obesity paradox in CRC are scarce. Therefore, we investigated the differences in gene expression, TIIC occurrence, and intratumoral microbiome composition according to BMI via bioinformatic analysis of CRC TCGA data.

We confirmed the obesity paradox in CRC for TCGA-COAD and TCGA-READ cohorts. As shown in Figure 1, high BMI was associated with a favorable prognosis, i.e., higher OS rates. The PPI network analysis of DEGs identified 18 and 21 genes with upregulated and downregulated expressions, respectively (Figure 2E-H). Many studies have reported the functions of histone variants in CRC 63, and various dysregulated genes related to CRC prognosis have also been reported: For example, high expression levels of *CXCR2*, *IGF1*, *IL1A*, *OSM*, and *PLA2G4A*, and hypermethylated *MAPT* were associated with poor prognosis 64-69; low expression levels of *GFAP* and *PLA2G2A* were associated with poor prognosis 70, 71. However, *ALOX15B* and *PTGS2* expression levels were not associated with CRC prognosis 72, 73. Although a more detailed analysis on the role of these aberrantly expressed genes is required, our findings suggest that these DEGs are collectively responsible for enhanced survival in the high-BMI group.

GO analysis of the DEGs revealed that glutathione transferase activity was high in the high-BMI group (Figure 3A). We also observed the existence of a correlation between *GSTA1* and resting CD4 T cells (Supplementary Figure S2). However, CRC prognosis was not dependent on TIIC (Supplementary Table S1). Therefore, we believe that GST activity affects the obesity paradox of CRC patients in different ways. Indeed, a meta-analysis showed that *GSTM1* and *GSTT1* null genotypes contributed to an increased risk of CRC in the Caucasian population 74. Low expression of *GSTM1* and *GSTM2* was associated with better prognosis of CRC 75. In addition, GST-pi serves as an effective marker of survival in CRC 76. These findings provide a strong foundation for the association of glutathione s-transferase (GST) activity with prognosis in CRC. Another study reported that high levels of GST activity were associated with better survival and prognosis in ovarian cancer 77. GST is considered to lower the risk of cancer by regulating reactive oxygen species (ROS) 78. In contrast, GO analysis showed that the expression of long-chain fatty acid pathways, including the AA pathway, were lower in the high-BMI CRC patient group. *In vitro* studies using human cell lines derived from lung cancer and CRC have shown that AA inhibitors induce apoptosis 79, 80. Disease-free survival of cholangiocarcinoma patients with low expression of the AA pathway-associated COX-2 and 5-LOX showed better prognosis 81. Inhibition of the AA pathway enzymes of COX-2, 5-LOX, and CYP450 could inhibit cell proliferation and neoangiogenesis 82. Oral cancer patients with asymptomatic loss-of-function somatic mutations in the AA pathway showed good response to chemotherapy, which was likely because of an associated downregulation of the PI3K-Akt pathway downstream 83. In contrast, dysregulation of the eicosanoid pathway by chronic inflammation has complex implications for tumorigenesis, i.e., both cancer-promoting and anti-cancer roles 84. A previous study revealed that obesity was positively associated with AA-derived 5- and 11-hydroxyeicosatetraenoic acid levels 85. Obesity induces increased AA metabolism and activates various signaling pathways, including the PI3K-Akt pathway, and inflammatory cytokines, which have conflicting effects on CRC progression. Therefore, the downregulation of long-chain fatty acid metabolic pathways because of obesity does not necessarily improve prognosis in CRC.

KEGG pathway analysis revealed that drug metabolism of cytochrome P450 and metabolism of xenobiotics by cytochrome P450 were up- and downregulated, respectively (Figure 3C and 3D). This is consistent with the PPI analysis wherein the expressions of *ADLH3A1*, *GSTA1*, *GSTM1*, and *HPGDS* were upregulated and those of *CYP2C9*, *CYP2E1*, and *UGT1A6* were downregulated in the high-BMI CRC patient group with good prognosis. As a matter of fact, obesity has been reported to increase the activity of cytochrome P450 2E1 86. Although more detailed mechanistic studies are required, these results suggest that cytochrome P450-related genes are critical to the obesity paradox of CRC.

TIICs are important determinants of tumor development and clinical outcomes in cancer patients 23. Increased levels of tumor-infiltrating Tfh cells were correlated with increased survival of melanoma cancer patients 87, and favorable prognosis in lung squamous cell carcinoma 88. Tfh cells are more abundant in obese than lean mice 89. However, in the present study, we identified higher levels of resting CD4+ T cells and lower levels of Tfh cells in the high-BMI group.

As shown in Supplementary Figures S2 and S3, *CNGA3*, *GSTA1*, *HPGDS*, *FRZB*, and *WNT4* were significantly correlated with resting CD4+ T cells, and *IL1RN*, *OSM*, and *PLA2G2A* were significantly correlated with Tfh cells. Wnt/β-catenin signaling plays an important role in T-cell immunity 90 and cancer immunotherapy 91. Moreover, Thf cells may be involved in the occurrence of immune-related adverse events in highly efficient immune checkpoint blockade treatment through exaggerating cytotoxic T lymphocyte response 92. The generation of robust memory T cell populations is critical for T cell-based therapies to prevent and treat cancer 93. In contrast, a previous study reported that OSM not only increases the metastatic potential of breast cancer *in vitro* but also promotes metastasis *in vivo*, and may negatively affect patient survival 94. Lin Wang et al. 95 showed that *OSM* is associated with CD4 T cells and high-infiltration of Tfh was associated with poor prognosis. Our finding may provide guidance for further investigations regarding the mechanism of the obesity paradox in CRC. A previous report found that high-BMI patients (>30 kg/m^2^) have higher levels of macrophage M1 (1.13-fold higher than the 25-18.5 kg/m^2^ group) and lower levels of activated natural killer cells (0.25-fold lower than the 25-18.5 kg/m^2^ group) 96. Even though in the aforementioned study, CIBERSORT with gene expression and clinical data corresponding to CRC patients from the TCGA database was used, their results were distinctly different from ours. However, we could not conduct an informed comparison as the description of their dataset was limited. These results suggest that a more in-depth study on the role of TIICs in the obesity paradox of CRC is required.

Gut microbiota plays a vital role in regulating tumorigenesis and the progression of CRC 97-99. A recent meta-analysis of CRC showed that high levels of *Fusobacterium nucleatum* and *Bacteroides fragilis* were related with poor and improved survival, respectively 100. Although we did not identify *F. nucleatum* or *B. fragilis*, *Bifidobacterium* was found in CRC samples of high-BMI patients. *Bifidobacterium* was previously found in the fecal samples of a healthy control group 101 and occurred at a low level in a CRC patient. Kosumi et al. 102 showed that the abundance of *Bifidobacterium* was associated with the level of signet ring cells, suggesting that *Bifidobacterium* might affect the tumor microenvironment and differentiation of cancer cells. Nevertheless, there was no significant difference in survival probability related to *Bifidobacterium*. Although little evidence exists for *Bifidobacterium* improving survival in CRC patients, the existing literature and our results suggest that *Bifidobacterium* is a potential diagnostic and prognostic marker for CRC. Recently, it has been reported that *Bifidobacterium lactis* and *Lactobacillus plantarum* suppress glioma growth in mice by inhibiting the PI3K-Akt pathway 103. Our results revealed that the AA pathway was lower but the KEGG enrichment analysis of the 14 upregulated-hypomethylated genes was associated with mTOR signaling pathway in the high-BMI CRC patients. Therefore, further studies are needed to investigate the effect and mechanisms of *Bifidobacterium* on the AA/PI3K-Akt/mTOR signaling pathway, and for this, a research model using CRC organoid needs to be considered.

We acknowledge that there are some limitations to our study. First, considering the aim of our study - the assessment of the molecular and prognostic differences between CRC patients of varying BMI - it is difficult to determine obesity based on BMI. As we used TCGA datasets, we had to apply the WHO standard of 30 kg/m^2^ to define obesity. However, considering the molecular changes caused by obesity, it may be more appropriate to use waist circumference rather than BMI; however, TCGA database does not provide waist circumference. Second, although the histological type of CRC is an important factor for prognosis, it was not stratified in TCGA data. Third, the immune cell infiltration assays and microbiome analysis were based on bioinformatic techniques. Finally, we did not examine the relationship between the molecular effects of obesity and sequential change of the microbiome and the feedback between these factors. Given these limitations, it is difficult to conclude the plausibility of the obesity paradox in CRC. Future research should consider the causal relationship or underlying mechanism of the obesity paradox in CRC.

## Conclusion

CRC is a common malignancy worldwide and is the second leading cause of cancer-related deaths. Obesity paradox is a phenomenon in which the prognosis of overweight patients is superior to that of underweight and normal weight patients in several chronic diseases such as CRC. This study shows that high-BMI patients with CRC have better prognosis, higher levels of resting CD4+ T cells, lower levels of Tfh cells, and different levels of intratumoral microbiota than low-BMI patients. Our study highlights the TIICs and intratumoral microbe diversity as major features of the obesity paradox in CRC.

## Supplementary Material

Supplementary figures and table.

## Figures and Tables

**Table 1:** Characteristics of colorectal cancer (CRC) patients (TCGA-COAD and TCGA-READ)

TCGA-CRC	N	Mean (SD)
Variable		
**Age (years)**	178	64.5 (12.6)
	*N*	*Percentage (%)*
**Sex**	178	
Female	93	52.2
Male	85	47.8
**Race**	178	
White	138	77.5
American Indian	1	0.6
Black	39	21.9
**Primary Disease Stage**	172	
I-II	94	54.7
III-IV	78	45.3
**Sample type**	178	
Primary	178	100
**BMI**	178	
≥ 30	88	49.4
≤ 25	90	50.6
**Death**	178	
No	144	80.9
Yes	34	19.1

CRC, colorectal cancer; BMI, body mass index; SD, standard deviation.

**Table 2:** Cox proportional hazard analysis of overall survival in colorectal cancer patients

Variable	HR^1^	95% CI^2^	*P*-value
**BMI**			
High (≥ 30)	reference		
Low (≤ 25)	2.49	1.06-5.9	0.037^*^
Age	1.03	1.00-1.1	0.071
**Sex**			
Female	reference		
Male	0.59	0.28-1.2	0.16
**Disease Stage**			
I-II	reference		
III-IV	3.44	1.58-7.5	0.002^*^

Global P-value (log-rank) = 0.0006^*^; Concordance index = 0.72; ^1^HR: adjusted hazard ratio, estimated using the Cox proportional hazards model. ^2^CI: confidence interval. ^*^P-value statistically significant.

## Abbreviations

BMI: body mass index
CRC: colorectal cancer
DEGs: differentially expressed genes
GO: gene ontology
KEGG: Kyoto encyclopedia of genes and genomes
LEfSe: linear discriminant analysis effect size
MCODE: Molecular Complex Detection
PPI: protein-protein interaction
TCGA: The Cancer Genome Atlas
Tfh: T follicular helper cells
TIIC: tumor-infiltrating immune cell
