# Supplementary material for: Bioinformatic analysis of the obesity paradox and possible associated factors in colorectal cancer using TCGA cohorts
Source: J Cancer. 2023 Jan 22;14(3):322–35. doi: 10.7150/jca.80977 (PMC9969588; doi:10.7150/jca.80977)
Supplement: Supplementary file 1 — Supplementary figures and table. [file jcav14p0322s1.pdf]

Supplementary Figures

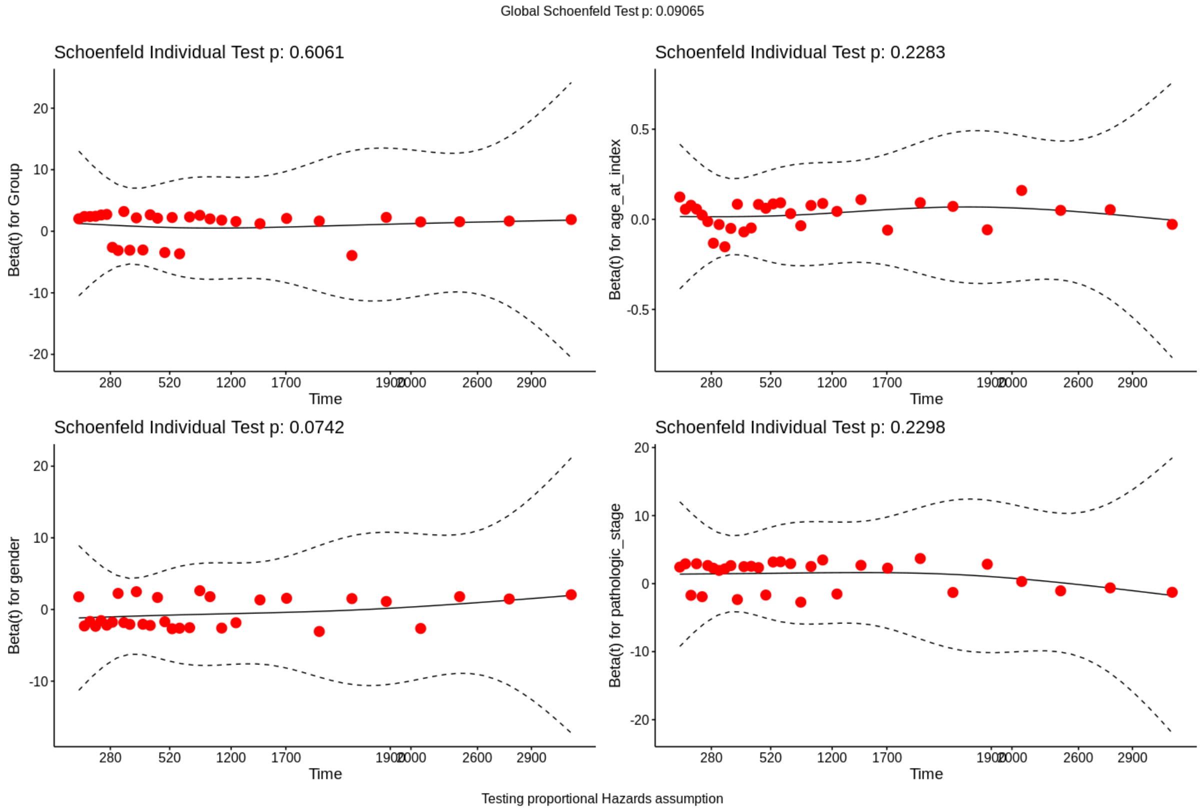

Supplementary Figure S1. Schoenfeld residual diagram. The curve of each diagram represents the trend of factor change as time goes by. The *P*-values of the variables and the model as a whole were shown in the diagram. *P*-value > 0.05 indicates that the variable meets the cox proportional hazard assumption.

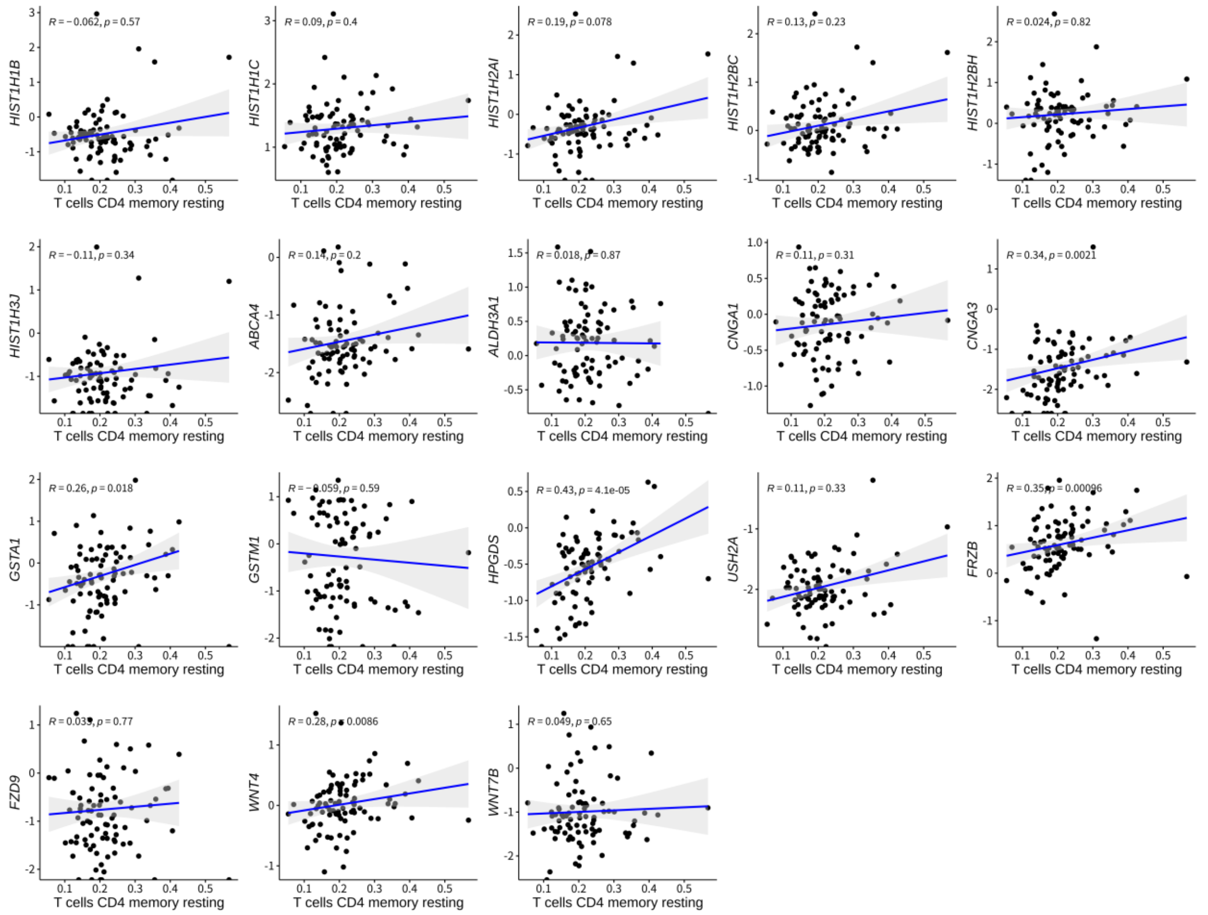

Supplementary Figure S2. Scatter plot of Spearman correlation analysis between 18 upregulated genes and resting CD4 T cells. The x-axis represents the cell fraction values, and the y-axis represents the normalized gene expression values of upregulated genes. Correlation coefficients and *P*-values are shown inside the plots.

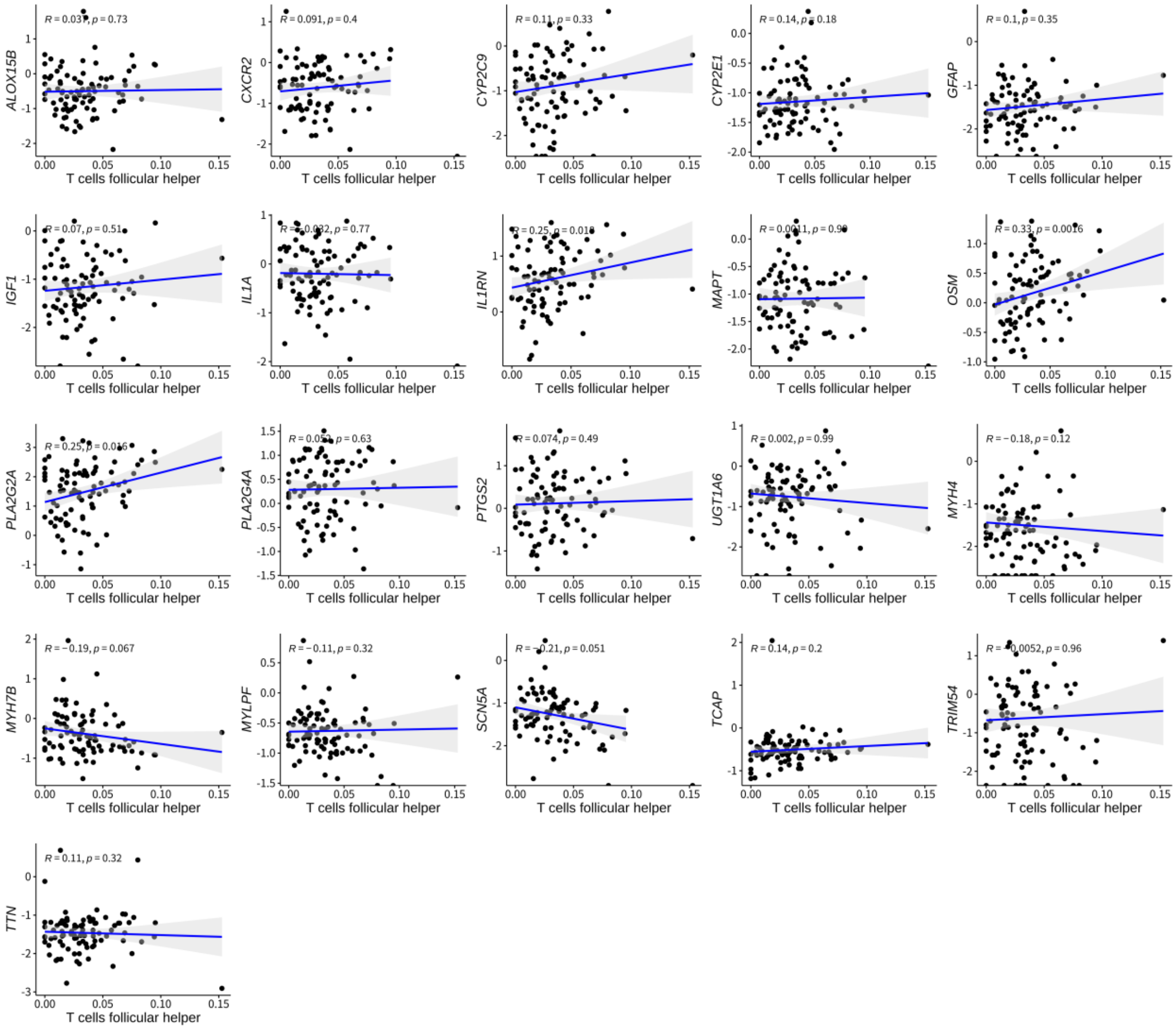

Supplementary Figure S3. Scatter plot of Spearman correlation analysis between 21 downregulated genes and Tfh cells. The x-axis represents the cell fraction value, and the y-axis represents the normalized gene expression value of downregulated genes. Correlation coefficients and *P*-values are shown inside the plots.

Supplementary Table S1. Univariate and multivariate analyses between BMI and TIICs in CRC patients.

Variable	Univariate analysis			Multivariate analysis		
	HR[1]	95% CI[2]	*P*-value	HR[1]	95% CI[2]	*P*-value
BMI	0.435	0.203 - 0.935	0.024*	0.520	0.215 - 0.215	0.147
B.cells.memory	1.714	0.870 - 3.376	0.122	1.915	0.855 - 0.855	0.114
B.cells.naive	1.134	0.569 - 2.259	0.721	1.214	0.462 - 0.462	0.694
Dendritic.cells.activated	0.505	0.249 - 1.024	0.052	0.552	0.214 - 0.214	0.219
Dendritic.cells.resting	1.287	0.650 - 2.549	0.469	1.260	0.529 - 0.529	0.602
Eosinophils	0.574	0.286 - 1.150	0.112	0.923	0.384 - 0.384	0.859
Macrophages.M0	1.448	0.733 - 2.860	0.286	0.734	0.259 - 0.259	0.560
Macrophages.M1	1.086	0.543 - 2.175	0.816	0.604	0.191 - 0.191	0.389
Macrophages.M2	1.626	0.815 - 3.244	0.167	2.350	0.683 - 0.683	0.176
Mast.cells.activated	0.498	0.247 - 1.004	0.047*	0.668	0.235 - 0.235	0.449
Mast.cells.resting	1.582	0.787 - 3.178	0.196	1.169	0.374 - 0.374	0.788
Monocytes	0.456	0.220 - 0.942	0.029*	0.649	0.236 - 0.236	0.402
Neutrophils	0.758	0.367 - 1.567	0.451	0.808	0.321 - 0.321	0.652
NK.cells.activated	1.015	0.516 - 1.995	0.966	1.083	0.450 - 0.450	0.860
NK.cells.resting	0.880	0.445 - 1.739	0.713	1.203	0.480 - 0.480	0.693
Plasma.cells	0.962	0.463 - 2.000	0.918	1.098	0.389 - 0.389	0.860
T.cells.CD4.memory.activated	0.894	0.451 - 1.771	0.747	0.803	0.337 - 0.337	0.620
T.cells.CD4.memory.resting	0.576	0.285 - 1.167	0.118	0.708	0.275 - 0.275	0.473
T.cells.CD4.naive	0.897	0.119 - 6.793	0.915	0.437	0.033 - 0.033	0.530
T.cells.CD8	1.376	0.696 - 2.719	0.358	1.540	0.504 - 0.504	0.449
T.cells.follicular.helper	0.996	0.506 - 1.958	0.990	0.873	0.318 - 0.318	0.791
T.cells.gamma.delta	1.309	0.589 - 2.912	0.519	2.030	0.730 - 0.730	0.175
Tregs	1.105	0.562 - 2.172	0.772	0.716	0.283 - 0.283	0.480

[1]HR: adjusted hazard ratio.

[2]CI: confidence interval.

**P*-value statistically significant
